# Muscle Damage following Maximal Eccentric Knee Extensions in Males and Females

**DOI:** 10.1371/journal.pone.0150848

**Published:** 2016-03-17

**Authors:** K. M. Hicks, G. L. Onambélé, K. Winwood, C. I. Morse

**Affiliations:** 1 Department of Sport, Exercise and Rehabilitation, Northumbria University, Newcastle-Upon-Tyne, United Kingdom; 2 Institute for Performance Research, Department of Exercise and Sport Science, Manchester Metropolitan University Cheshire, Manchester, United Kingdom; West Virginia University School of Medicine, UNITED STATES

## Abstract

**Aim:**

To investigate whether there is a sex difference in exercise induced muscle damage.

**Materials and Method:**

*Vastus Lateralis* and patella tendon properties were measured in males and females using ultrasonography. During maximal voluntary eccentric knee extensions (12 reps x 6 sets), *Vastus Lateralis* fascicle lengthening and maximal voluntary eccentric knee extensions torque were recorded every 10° of knee joint angle (20–90°). Isometric torque, Creatine Kinase and muscle soreness were measured *pre*, *post*, *48*, *96 and 168* hours post damage as markers of exercise induced muscle damage.

**Results:**

Patella tendon stiffness and *Vastus Lateralis* fascicle lengthening were significantly higher in males compared to females (p<0.05). There was no sex difference in isometric torque loss and muscle soreness post exercise induced muscle damage (p>0.05). Creatine Kinase levels post exercise induced muscle damage were higher in males compared to females (p<0.05), and remained higher when maximal voluntary eccentric knee extension torque, relative to estimated quadriceps anatomical cross sectional area, was taken as a covariate (p<0.05).

**Conclusion:**

Based on isometric torque loss, there is no sex difference in exercise induced muscle damage. The higher Creatine Kinase in males could not be explained by differences in maximal voluntary eccentric knee extension torque, *Vastus Lateralis* fascicle lengthening and patella tendon stiffness. Further research is required to understand the significant sex differences in Creatine Kinase levels following exercise induced muscle damage.

## Introduction

It is well established that unaccustomed eccentric exercise causes exercise induced muscle damage (EIMD). Although there remains no conclusion on the initial events leading to EIMD (direct damage to sarcomeres, or the Excitation-Contraction coupling process), the high stress and strain of eccentric contractions are associated with subsequent increases in markers of EIMD [1,2]. The degree of EIMD has been quantified using both direct (e.g. muscle biopsies) and indirect techniques [3,4]. Due to the invasive procedure and the small sampling area of muscle biopsies however, indirect techniques are more common [4]. A reduction in maximal isometric torque following EIMD has been reported as the most valid indirect marker of EIMD [3]; although, a change in range of motion, muscle soreness, and the detection of intramuscular enzymes (e.g. Creatine Kinase (CK)) in venous blood, are frequently reported as indirect markers of EIMD [3].

Within animals, EIMD is reported to be significantly higher in males compared to females [5]. Within human research however, the evidence is not as compelling with findings that support [6–10], or refute [11–14] sex differences in EIMD. In support of no sex difference in EIMD, Stupka, Lowther (12) showed that following repeated maximal leg press and leg extensions, there were similar amounts of Z-line streaming and CK between males and females. Whereas, Joyce, Sabapathy (9) and Sewright, Hubal (7) reported CK to be significantly higher in males compared to females following a high volume of eccentric contractions. In contrast, following a 30 minute single leg eccentric step exercise protocol, Fredsted, Clausen (10) reported CK and maximal torque loss to be significantly lower in males compared to females. The aforementioned discrepancies may be attributed to differences in exercise volume [15] and/or different muscle architecture and tendon properties, for example in the elbow flexors compared to the knee extensors [16].

Previous literature has alluded to the tendon potentially acting as a mechanical buffer during eccentric contractions [17,18]. Using Hill’s three element muscle model, Lichtwark and Wilson [19] found during the stance phase of walking, increasing Achilles tendon stiffness augmented fascicle lengthening despite total muscle tendon unit lengthening remaining constant. In agreement, we [20] have previously attributed greater *Vastus Lateralis* fascicle lengthening during a single bout of eccentric contractions in males, to a significantly higher Young’s Modulus of the patella tendon compared to females. Thus the males experienced significantly higher levels of fascicle lengthening, which, as a determinant of EIMD *in vitro* (albeit in animals [2]), may explain higher levels of EIMD previously reported in males. However, any sex difference in fascicle lengthening, tendon properties and EIMD *in vivo* remains to be confirmed experimentally.

Within humans a sex difference in EIMD remains equivocal, furthermore, a physiological explanation for the potential differences has yet to be reported. Therefore, the aims of this current study were twofold: firstly, to establish whether there is a sex difference in EIMD, when difference in muscle size is accounted for. Secondly, dependent on the outcome of the first aim, to establish whether tendon stiffness may explain the potential sex difference in EIMD.

## Materials and Methods

### Subjects

11 males (21.1 ± 1.6 years of age, 72.0 ± 7.5 kg and 176 ± 6 cm) and 11 females (21.4 ± 1.6 years of age, 63.0 ± 5.8 kg and 165 ± 8 cm) signed written informed consent to participate in this study. All participants self-reported as being recreationally active (undertaking no more than 1 hour of “moderate” physical activity per week) and did not take part in any structured resistance training. None of the female participants had ever used any form of oestrogen-based contraception. All women reported regular menstrual cycles, documenting an average cycle length of 28 ± 1 days. Females were tested on the 14^th^ day (self-reported) of the menstrual cycle to measure oestrogen levels at ovulation [21]. All procedures complied with the Declaration of Helsinki and ethical approval was obtained through the local ethics committee board at Manchester Metropolitan University [22]. Exclusion criteria included any resistance training in the last six months, occupation or lifestyle that required regular heavy lifting or carrying, any known muscle disorder, the use of dietary supplements (i.e. vitamin e), and any musculoskeletal injury in the last three months. Further exclusion criteria for female participants included, irregular menstrual cycles (where regular cycles were defined as 24–35 days) in the last 12 months, and pregnant in the year preceding inclusion in the present study. All inclusion and exclusion criteria were determined through participant questionnaire prior to inclusion within this study.

### Testing protocol

Participants attended the laboratory on five different occasions over nine days. The sessions were as follows: 1) *pre damage* 2) *damage*, 3) *48 hours*, 4) *96 hours*, and 5) *168 hours*. *Pre damage* assessments consisted of stature and mass (anthropometric measures), patella tendon moment arm, 5–6ml blood sample, dynamometer familiarization (within *pre damage*), morphological measures of the patella tendon (tendon size and stiffness) and maximal voluntary isometric knee extension torque measurements at six knee angles (60, 65, 70, 75, 80 and 90° (0° = full extension). Participants were tested at six knee angles to obtain optimal knee angle for maximal voluntary isometric knee extension torque. Participants only performed two practice isometric maximal voluntary contractions, at two knee joint angles during the familiarization session. Stature and mass were measured using a wall mounted stadiometer (Harpenden, Holtain Crymych, UK) and digital scales (Seca model 873, Seca, Germany) respectively. The *damage* session consisted of maximal voluntary eccentric knee extensions, 5–6 mL venous blood sample, rating of muscle soreness and maximal voluntary isometric knee extension torque measurements. The *48*, *96 and 168 hours session* consisted of ~5–6 mL blood sample, rating of muscle soreness, and maximal voluntary isometric knee extension torque measurements at all six knee angles (60, 65, 70, 75, 80 and 90°).

All tests were carried out in the non-dominant leg. The non-dominant leg was defined as the leg that provided stability during movements, e.g. kicking. Participants were seated in an isokinetic dynamometer (Cybex Norm, Cybex International, NY, USA), with a hip angle of 85° (0° = supine). To reduce any extraneous movement during maximal efforts participants were secured in a seated position using inextensible straps around the shoulders and hips. The isokinetic dynamometer axis of rotation was visually aligned with the knee joint’s center of rotation. During *pre damage* the isokinetic dynamometer settings and anatomical zero were recorded to ensure repeatability in the following sessions.

#### Vastus Lateralis anatomical cross-sectional area

*Vastus Lateralis* anatomical cross-sectional area (VL_ACSA_) was measured using a real-time B-mode ultrasound (AU5 Harmonic, Esaote Biomedica, Genoa, Italy). With the participant laid supine and their non-dominant leg fully extended (knee angle 0°), the distal and proximal insertions sites of the *Vastus Lateralis* were identified using an ultrasound probe (7.5 MHz linear array probe, 38 mm wide). At 50% of *Vastus Lateralis* muscle length echo-absorptive markers were placed in parallel, at intervals of 30 mm, from the lateral to the medial edge of the *Vastus Lateralis* muscle. The ultrasound probe was held perpendicular to the *Vastus Lateralis* muscle in the axial plane. The ultrasound probe was moved steadily over the echo-absorptive markers from the lateral to the medial edge of the muscle. Constant, light pressure was placed on the muscle during scanning to avoid compression of the muscle. The images were recorded in real time at 25 frames per second (Adobe Premier pro Version 6, Adobe Systems Software, Ireland). Using video capture software (Adobe Premier Elements, version 10), individual images were acquired at each 30 mm interval. Shadows cast by the echo-absorptive markers allowed the images to be aligned by the outline of the muscle, thus forming the entire VL_ACSA_ in a single image (Adobe Photoshop Elements, version 10). Digitizing software (ImageJ 1.45, National Institutes of Health, USA) was used to measure VL_ACSA_. This method of calculating VL_ACSA_ has previously been accepted as reliable and valid when compared to MRI, with a reported interclass correlation between 0.998 and 0.999 [23].

Within males a VL_ACSA_ of 24.3 cm^2^ has previously reported to contribute to ~32% of total quadriceps anatomical cross sectional area (Q_ACSA_ = 74.9 cm^2^ [24]). Where in females, a VL_ACSA_ of ~21 cm^2^ has been reported to contribute to 38% of Q_ACSA_ (Q_ACSA_ = ~55 cm^2^ [25]). Therefore, assuming the contribution of VL_ACSA_ remains constant within males and females, Q_ACSA_ within males and females was estimated by multiplying VL_ACSA_ by 3.08 and 2.61, respectively.

#### Torque measurements

At six different knee angles (60, 65, 70, 75, 80 and 90° (full extension = 0°)) participants were instructed to perform two maximal voluntary isometric knee extensions lasting ~two seconds with 90 seconds rest between contractions. Torque was presented, in real time, on a Macintosh G4 computer (Apple Computer, Cupertino, CA, USA), via an A/D converter (Biopac Systems, Santa Barbara, CA). Torque measurements were later analyzed offline with the accompanying software (Acqknowledge, version 3.9.2). The highest torque produced at each angle was taken as maximal voluntary isometric knee extension peak torque. The angle where the highest maximal voluntary isometric knee extension torque was produced during the pre session was recorded as optimal knee angle. Optimal knee angle was used to calculate maximal voluntary isometric knee extension torque loss in the subsequent sessions. To calculate loss of maximal voluntary isometric knee extension torque following eccentric exercise, maximal voluntary isometric knee extension torque measurements were repeated 60 minutes post eccentric exercise (to reduce any fatigue effect [26]) and *48 hours*, *96 hours* and *168 hours* (recovery) post eccentric exercise.

#### Patella tendon length and cross-sectional area

**Fig 1:**
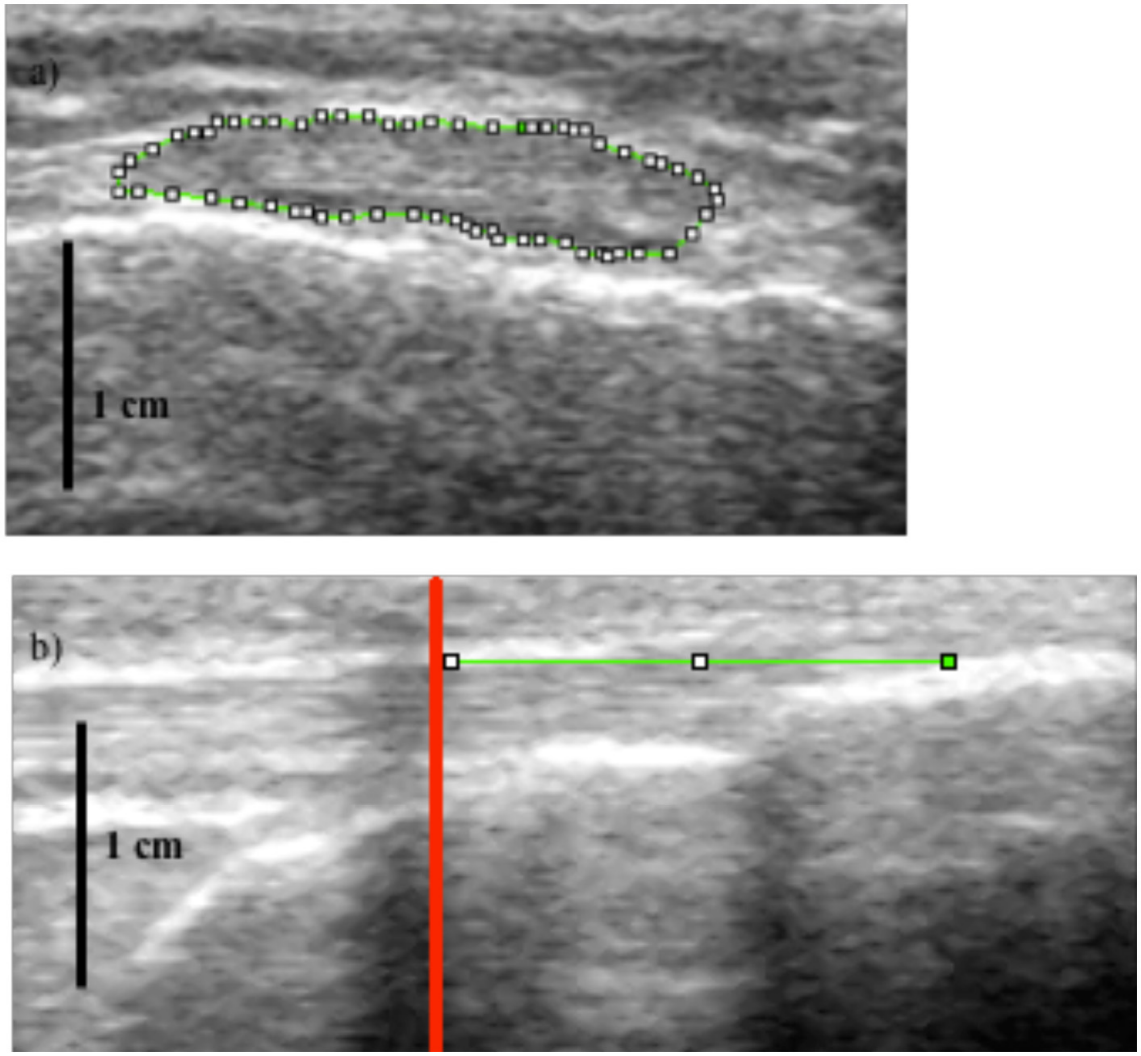
a) Example figure of measuring patella tendon cross-sectional area at 50% of patella tendon length using ultrasound. b) Example figure of measuring patella tendon lengthening using ultrasound. The red line emphasizes the shadow cast by the echo-absorptive marker on the skin, with the displacement of the proximal or distal anatomical marker made relative to this line. The distal insertion of the patella tendon into the tibia is shown in (b).

A real-time B-mode ultrasound (AU5 Harmonic, Esaote Biomedica, Genoa, Italy) was used to measure patella tendon cross-sectional area and patella tendon length at a fixed 90° knee angle. For measuring patella tendon length, the location of the apex of the patella tendon and the superior aspect of the tibial tuberosity were marked using sagittal-plane ultrasound images. The distance between the apex of the patella tendon and the superior aspect of the tibial tuberosity was then measured to calculate patella tendon length. To measure patella tendon cross-sectional area, the ultrasound probe was placed in the transverse plane and images were captured at 25%, 50%, and 75% of patella tendon length (Fig 1a). The images were later analyzed offline using ImageJ (1.45, National Institutes of Health, USA). A mean of all three images were taken for patella tendon cross-sectional area.

#### Patella tendon stiffness

The participants were seated in the isokinetic dynamometer, with the knee angle fixed at 90°. Firstly, to obtain peak maximal voluntary isometric knee extension, participants were instructed to perform two maximal voluntary isometric knee extensions lasting ~two seconds with 90 seconds rest between contractions. Consistent with previous studies [27], and the tracking of the patella tendon for the assessment of patella tendon stiffness participants were instructed to reach the peak maximal voluntary isometric knee extension torque, by performing a ramped maximal voluntary isometric knee extension lasting ~5–6 seconds. Ramped maximal voluntary isometric knee extension torque and displacement of the patella tendon were synchronized using a 10-V square wave signal generator. Patella tendon displacement was measured over two ramped maximal voluntary isometric knee extensions, once with the ultrasound probe positioned over the distal edge of the patella and on the second contraction over the tibial tuberosity [27], so that total displacement would be computed from the composite of proximal and distal patella motions (see below). Torque was presented on a Macintosh G4 computer (Apple Computer, Cupertino, CA, USA), via an A/D converter and subsequently analyzed with the accompanying software (Acknowledge, Biopac Systems, Santa Barbara, CA). To create an external marker on the ultrasound images, an echo-absorptive marker was placed on the skin. Using the marker to calculate displacement of the tendon, the distance of the marker (shadow) from an anatomical reference point at the beginning of the contraction, to the position of the shadow at the end of the contraction was calculated (Fig 1b). A 10-V square wave signal generator was used to synchronize the ultrasound images with the torque acquisition system. Images were captured at ~10% intervals of ramped maximal voluntary isometric knee extension torque [27]. Total patella tendon displacement was calculated as displacement at the apex of the patellar plus the displacement at the tibial tuberosity [27]. Patella tendon forces were calculated as: (maximal voluntary isometric knee extension torque + antagonist co-activation torque) / patella tendon moment arm.

Patella tendon moment arm was measured at 90° (full extension = 0°) in the sagittal plane, from a dual-energy X-ray absorptiometry scan (frame 23.3 cm x 13.7 cm, Hologic Discovery, Vertec Scientific Ltd, UK), and subsequently analyzed using a DICOM image assessment tool (OsiriX DICOM viewer, ver. 4.0, Pixemo, Switzerland). Patella tendon moment arm length was determined as the perpendicular distance from the center of the patella tendon to the tibio–femoral contact point. Dual-energy X-ray absorptiometry scans have been compared to MRI measures, demonstrating consistent reliability and validity against this standard [28].

To compute tendon forces, the calculation of antagonist co-activation torque is described below. The force—patella tendon elongation curve stemming from data at every 10% of maximal voluntary isometric knee extension was then fitted with a second-order polynomial function forced through zero [27]. The tangential slope at discreet sections of the curve, relative to maximal voluntary isometric knee extension force, was computed by differentiating the curve at every 10% force intervals. In addition, to standardize the comparison of tendon stiffness at an absolute load, the slope of the tangential line, corresponding to the maximal voluntary isometric knee extension force of the weakest participant, was computed for each subject.

#### Co-activation

To determine co-activation during the ramped maximal voluntary isometric knee extension, Electromyogram (EMG) of the long head of the *bicep femoris* was measured. Guided by an axial-plane ultrasound of the long head of the *bicep femoris*, two bipolar electrodes (Ambu, Neuroline 720, Denmark) were placed in the mid-sagittal line at 25% of *bicep femoris* muscle length (distal end = 0%). A reference electrode (Ambu, Blue Sensor, Denmark) was placed on the lateral tibial condyle. The electrodes were placed in a bipolar configuration with a constant inter-electrode distance of 20 mm. Prior to electrode placement; the skin was shaved, gently abraded and cleansed with an alcohol wipe to reduce skin impedance below 5000 Ω. To minimize cross talk, and ensure a mid-sagittal placement of electrodes, ultrasonography was used to identify the medial and lateral aspects of the *bicep femoris* muscle. The raw EMG signal was amplified (×2000) and filtered (through low and high pass filters of 10 and 500 Hz respectively) with the sampling frequency set at 2000 Hz. Ramped maximal voluntary isometric knee extension torque and *bicep femoris* EMG were recorded in real time and synchronized using an external square wave signal generator. Participants performed two maximal voluntary isometric knee flexions at 90° knee angle. They were instructed to perform maximal voluntary isometric knee flexions rapidly and as forcefully as possible against the dynamometer’s lever arm. The participants were instructed to relax once a two second plateau had been attained (as observed on the dynamometer screen display). The integral of the root mean square of the *bicep femoris* EMG signal, was calculated 500 ms either side of instantaneous maximal voluntary isometric knee flexions maximal torque from the contraction corresponding to the highest maximal voluntary isometric knee flexion torque. Prior to contraction the baseline signal noise was calculated as the integral root mean square over 1s and removed from the measured EMG during maximal voluntary isometric knee flexion and maximal voluntary isometric knee extension. At every 10% of ramped maximal voluntary isometric knee extension torque the absolute integral of the *bicep femoris* EMG was taken over 250 ms. Co-activation torque was calculated as, (*bicep femoris* EMG during ramped maximal voluntary isometric knee extension / *bicep femoris* EMG during maximal voluntary isometric knee flexion) × peak maximal voluntary isometric knee flexion torque at 90° knee angle [27]. This equation assumes that the *bicep femoris* is representative of the entire hamstring [29], and that a linear relationship exists between *bicep femoris* EMG and maximal voluntary isometric knee flexion torque [30].

#### Patella tendon stress / strain relationship

Patella tendon strain was calculated as a ratio of total patella tendon displacement and patella tendon length. Patella tendon stress was calculated as: patella tendon force (N) / patella tendon cross-sectional area (mm^2^).

#### Young’s modulus

Young’s modulus was calculated as, patella tendon stiffness × (patella tendon length (mm) / patella tendon cross-sectional area (mm^2^)).

#### ‘Damaging’ Eccentric exercise

Prior to eccentric exercise, a warm-up of 10 isokinetic knee extensions and knee flexions were carried out through the full test range of motion (20–90°, at 60°·s^-1^), ensuring a progressive increase in effort (with the last contraction being maximal). For the eccentric exercise, the knee extension range of motion was set at 20–90° (0° = full extension). Participants were asked to perform six sets of 12 maximal voluntary eccentric knee extensions, which has previously been reported to induce significant EIMD [31]. The eccentric phase of the contractions was performed at an angular velocity of 30°·s^-1^ [31]. The concentric phase was performed sub-maximally at an angular velocity of 60°·s^-1^ to minimize fatigue and enhance eccentric damage [32]. In a subpopulation of participants (n = 6) the concentric phase throughout the damaging protocol was measured at the angle consistent with peak maximal voluntary contraction torque. Values for concentric torque remained below 25% of maximal voluntary contraction, therefore at least nominally we refer to the EIMD as “Eccentric” rather than “Stretch-shortening” in nature. This keeps our terminology (and implementation) of isokinetic eccentric contraction consistent with previous. Two minutes’ rest was provided between each set. Participants remained seated in the isokinetic dynamometer throughout the entire exercise protocol, including rest periods. Visual feedback and verbal encouragement was continuously provided throughout the protocol. Maximal voluntary eccentric knee extension torque was recorded throughout each contraction and displayed via the torque acquisition system. For each set, peak maximal voluntary eccentric knee extension toque was determined as the highest torque out of the 12 repetitions. Average peak maximal voluntary eccentric knee extension torque was calculated as an average of peak maximal voluntary eccentric knee extensions across six sets.

#### Change in Vastus Lateralis fascicle length during the eccentric protocol

A real-time B-mode ultrasound (AU5 Harmonic, Esaote Biomedica, Genoa, Italy) was used to measure *Vastus Lateralis* fascicle length during maximal voluntary eccentric knee extensions. The ultrasound probe (7.5 MHz linear array probe) was fixed at 50% of *Vastus Lateralis* muscle length in the mid-sagittal plane of the non-dominant leg. A hypo-allergenic ultrasound gel (Parker, Park Laboratories Inc., Fairfield) was used to enhance acoustic coupling between the skin and the ultrasound probe.

During the first set (out of six) of maximal voluntary eccentric knee extension contractions, ultrasound images were recorded onto a PC, in real time, at 25 frames per second (Adobe Premier pro Version 6). An externally generated square wave signal was used to synchronize the ultrasound images with the torque acquisition system. Three maximal voluntary eccentric knee extension contractions were chosen from the first set of 12 repetitions for architectural analysis. Using frame capture software (Adobe Premier Elements, version 10), an ultrasound image (frame corresponding to every 10° of knee angle, ranging from 20–90°) was acquired for offline analysis using ImageJ (1.45, National Institutes of Health, USA). To ensure there was no movement artefact included in the measurement of fascicle length, an echo-absorptive marker was fixed on the skin to provide a visual reference point for the internal structures. If movement of the reference line was observed, the contraction was discarded and another repetition was chosen for analysis.

Using digitizing software (ImageJ 1.45, National Institutes of Health, USA), *Vastus Lateralis* fascicle length was analyzed offline at every 10° knee angle (range 20–90°, 0° = full extension) throughout the maximal voluntary eccentric knee extension. Fascicle length was measured from the visible insertion of the fibre into either the deep and superficial aponeurosis [33]. Where the fascicle extended longer than the ultrasound image (frame width 3.50 cm and height 4.15 cm), linear continuation of the fascicle and aponeurosis was assumed. Using ultrasound a 2–7% error has been associated with assuming linear continuation to calculate *Vastus Lateralis* fascicle length at 120° knee angle [34]. To reduce error associated with the estimation of *Vastus Lateralis* fascicle length, an average of three fascicles across the image was taken. Change in fascicle length is presented as fascicle length at a knee angle of 90° made relative to fascicle length measured at a knee angle of 20°; hereafter termed “relative fascicle lengthening” and reported as a percentage change from starting length at 20°.

#### Muscle soreness

Muscle soreness was measured using a visual analogue scale. The visual analogue scale consisted of a line which was 100 mm long, with 0 mm labelled and denoting “No pain at all” and 100 mm labelled and denoting “Unbearable pain”. Seated in the isokinetic dynamometer the non-dominant leg was passively moved through a full range of motion at 30°·s^-1^. Participants were asked to mark the visual analogue scale, between 0 mm–100 mm, to denote the level of pain they experienced during the passive movement. The visual analogue scale has been reported to be a valid and reliable measure of muscle soreness (Inter class correlation > 0.96 [35]).

#### Blood samples

Venous blood samples were taken to measure CK levels. A 21-gauge needle was inserted into the antecubical vein of the forearm, using a 10 mL syringe. Approximately 5–6 mL of blood was drawn into a serum collection tube. The sample was left on a crushed ice bed for 60 minutes. The sample was then centrifuged at 4500 rpm at 0°C for 10 minutes. Using a 200–1000 μl pipette (Eppendorf), the resulting serum sample was separated into three aliquots (~500 μl each) and stored in eppendorfs at -20°C until CK analysis was performed. Creatine kinase levels were measured using standard enzyme-linked immunosorbent assay (ELISA) procedures, measured at optimal density 340 nm (BioTek ELx800 96 well Microplate Reader) and immediately analyzed (Gen5, version 2.0). Each sample was run in duplicate-quadruplets using an EnzyChromTM CK Assay Kit (BioAssay Systems, Hayward, CA, sensitivity 5 U/L, intra-assay variability < 5%). An average of two—four readings was taken. CK activity is reported in absolute and absolute change ((ΔCK_ABS_) i.e. the peak CK value—the pre CK values) terms. Although CK is used as a marker of EIMD and does represent an extracellular expression of an intracellular protein [3], it is not possible to decipher whether an increase is due to a change in cell membrane permeability or structural damage [36]. Therefore, data from CK will be considered as a quantitative measure of damage with the caveat in mind that any difference between sex may not allow for a distinction to be made in whether this difference is due to a difference in membrane stability or contractile disruption.

### Statistics

Statistical analyses were carried out using the statistical software package SPSS (v.19, Chicago, IL) for Windows and Microsoft Excel. To ensure the data were parametric, the Levene’s and Shapiro-Wilk tests were used to assess the variance and normality of the data. If parametric tests were violated, the equivalent non-parametric tests were used. For sex differences in anthropometric measures, VL_ACSA_, Q_ACSA_ and patella tendon properties, independent T-tests and Mann-Whitney U tests were used. A 2×5 mixed design analysis of variance (ANOVA) (between factors: sex (2 levels), and time from EIMD (5 levels)) was used for muscle soreness, torque loss and CK levels. Wherever the assumption of sphericity was violated, the Greenhouse-Geisser correction was used. When a significant group effect was found an independent T-tests was used (planned contrast) with LSD correction. Since an association was reported between change in CK levels and maximal voluntary eccentric knee extensions normalized to Q_ACSA_, an analysis of covariance was used. Using G* Power (software version 3.1.9.2, University of Kiel, Germany) Cohen’s *d* was calculated for maximal voluntary isometric knee extension torque loss and peak CK. Significance was set at p≤ 0.05. Data are presented as mean ± standard deviation.

## Results

### Anthropometric measurements

There was no significant difference in age between males and females (p = 0.326). Males had significantly greater mass (p = 0.009) and were taller (p = 0.007) than females. Pre damage peak maximal voluntary isometric knee extension torque was significantly higher in males compared to females (263 ± 27 Nm, 190 ± 33 Nm, respectively, p = 0.0004). Other dimension and functional characteristics of the population are described in Table 1.

**Table 1:** Patella tendon properties in males and females.

	Males	Females
Patella tendon length (mm)	56.4 ** ± 5.05	48.3 ± 6.01
Patella tendon cross-sectional area (mm^2^)	83.9*** ± 16.4	54.2 ± 16.0
Patella tendon moment arm (cm)	4.41*** ± 0.31	3.90 ± 3.90
Ramped maximal voluntary isometric knee extension(Nm)	203*** ± 23	129 ± 26
Patella tendon force (N)	4707*** ± 603	3385 ± 700
Patella tendon stiffness (N·mm^·1^)	1387*** ± 560	610 ± 146
Young’s modulus (MPa)	948* ± 492	620 ± 284

**Note:** Patella tendon stiffness and Young’s modulus calculated at 100% maximal voluntary isometric knee extension,

* p<0.05,

** p<0.01,

*** p<0.0001.

Data is presented as means ± SD.

### VL_ACSA_ properties

VL_ACSA_ was 19.6% higher in males compared to females (24.4 ± 3.9 cm^2^ and 20.4 ± 3.4 cm^2^ respectively, p = 0.019). Estimated Q_ACSA_ was 26.0% higher in males compared to females (72.8 ± 14.0 cm^2^ and 53.9 ± 8.51 cm^2^ respectively, p = 0.0001).

### Tendon properties

Patella tendon properties for males and female are presented in Table 1. Patella tendon length was 14% longer in males compared to females (p<0.05). Patella tendon cross-sectional area was 39% larger in males (p<0.05). Ramped maximal voluntary isometric knee extension was 36% higher in males compared to females (P<0.05). Patella tendon moment arm length was 12% longer in males compared to females (p = 0.0007). Tendon stiffness and Young’s modulus at maximal voluntary isometric knee extension was 54% and 35% (respectively) higher in males compared to females. To account for the significantly higher ramped maximal voluntary isometric knee extension torque in males, the patella tendon force corresponding to the highest ramped maximal voluntary isometric knee extension torque of the weakest participant (independent of sex) was used to calculate standardized patella tendon stiffness at a standardized force (2330 N). Standardized patella tendon stiffness (males 998 ± 329 N·mm^·1^, females 542 ± 119 N·mm^·1^, p = 0.003) remained significantly higher in males compared to females.

### Torque production during maximal voluntary eccentric knee extensions

Average peak maximal voluntary eccentric knee extension torque of each set (six in total) was significantly higher in males compared to females (p = <0.05). Peak maximal voluntary eccentric knee extension torque averaged over six sets was significantly higher in males compared to females (255 ± 50 Nm and 167 ± 29 Nm, respectively, p = 0.0001). When peak maximal voluntary eccentric knee extension torque was normalized to Q_ACSA_, there was no significant difference between males and females (3.40 ± 0.78 Nm·cm^2^ and 3.15 ± 0.66 Nm·cm^2^, respectively, p = 0.160).

Peak maximal voluntary eccentric knee extension torque relative to pre damage maximal voluntary isometric knee extension torque, was not significantly different between males and females (97 ± 17% and 87 ± 12% respectively, p = 0.139).

### Vastus Lateralis fascicle length

Fascicle length at 20° knee joint angle was not significantly different between males and females (6.86 ± 0.31 cm, 6.67 ± 0.75 cm respectively, p = 0.197). The increase in fascicle length from 20° to 90° knee angle during maximal voluntary eccentric knee extension was 36% higher in males compared to females (3.84 ± 0.92 cm, 2.82 ± 0.45 cm, respectively, p = 0.007). *Vastus Lateralis* fascicle length at 90° knee angle made relative to fascicle length at knee angle of 20°, was significantly greater in males compared to females (56.0 ± 13.6% and 43.7 ± 10.1%, respectively, p = 0.034).

### Change in optimal knee angle

Pre-damage, optimal maximal voluntary isometric knee extension knee angle was not significantly different between males and females (median, 75.0 ± 10.0°, and 75.0 ±. 10.0°, respectively, p = 0.458). Post EIMD both males (90.0 ± 5.0°, p = 0.002) and females (median 80.0 ± 5.0°, p = 0.007) demonstrated a significant rightward shift in maximal voluntary isometric knee extension optimal angle. There was no significant difference in the magnitude of the rightward shift at the maximal voluntary isometric knee extension optimal angle between males and females (p = 0.099).

### Torque loss

**Fig 2:**
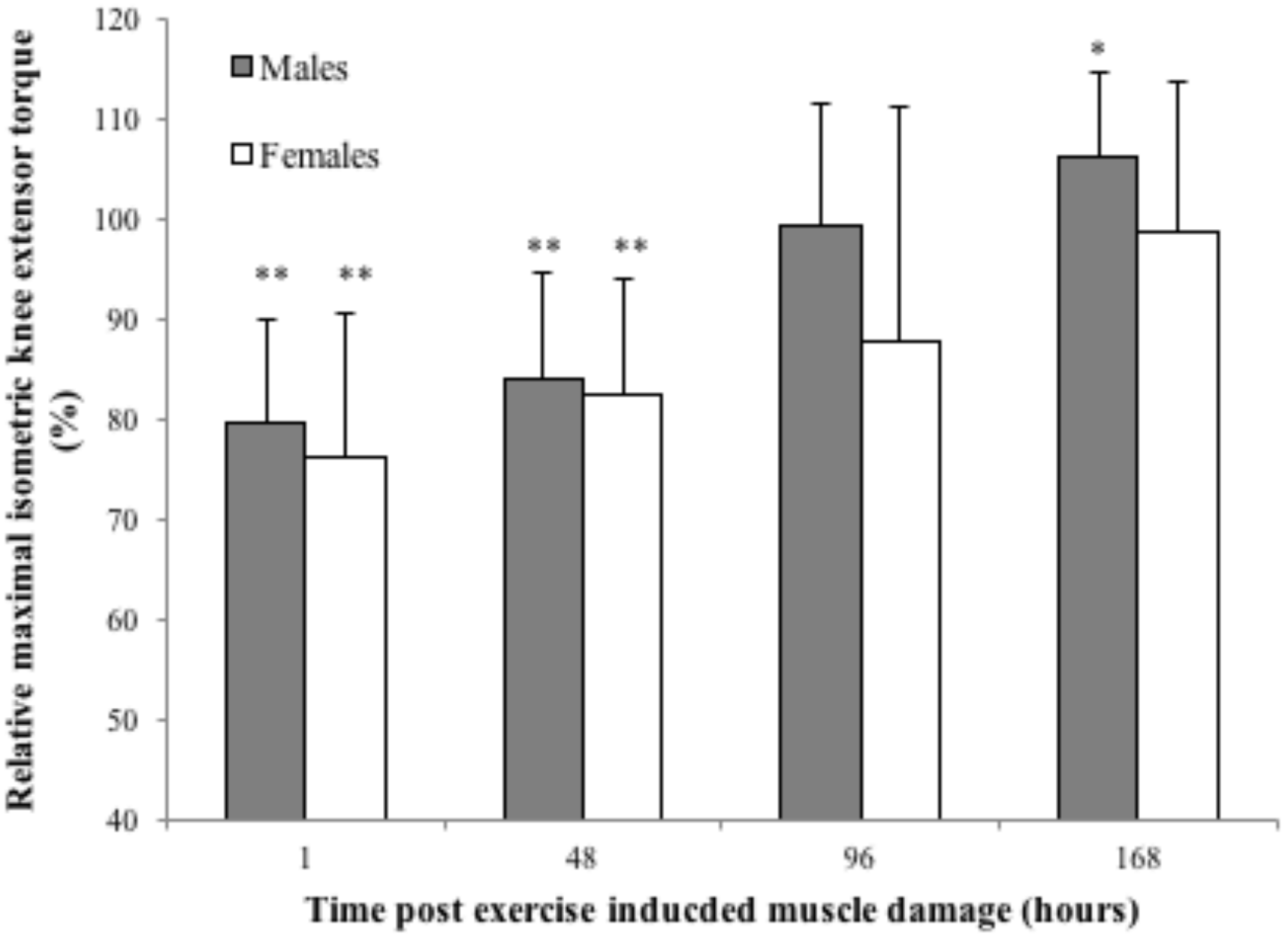
Maximal voluntary isometric knee extension torque loss, one, 48, 96 and 168 hours post exercise induced muscle damage (EIMD) in males and females. Maximal voluntary isometric knee extension torque loss is expressed as a percentage of maximal voluntary isometric knee extension torque pre exercise induced muscle damage. Data is presented as means ± SD. Significant difference from Pre–damage * p <0.05, ** p < 0.01.

Maximal voluntary isometric knee extension torque loss expressed as a percentage of pre-damage maximal voluntary isometric knee extension torque (males 263 ± 27 Nm and females 190 ± 33 Nm) is illustrated in Fig 2. A two-way mixed model ANOVA for maximal voluntary isometric knee extension torque loss reported a significant main effect of time (p = 0.0005), however there was neither a sex effect (p = 0.201) nor a time × sex interaction (p = 0.324). There was no significant difference in peak maximal voluntary isometric knee extension torque loss between males and females (22.5 ± 8.5% and 27.1 ± 13.1% respectively, p = 0.332, Cohen’s *d* = 0.44).

### CK levels

**Fig 3:**
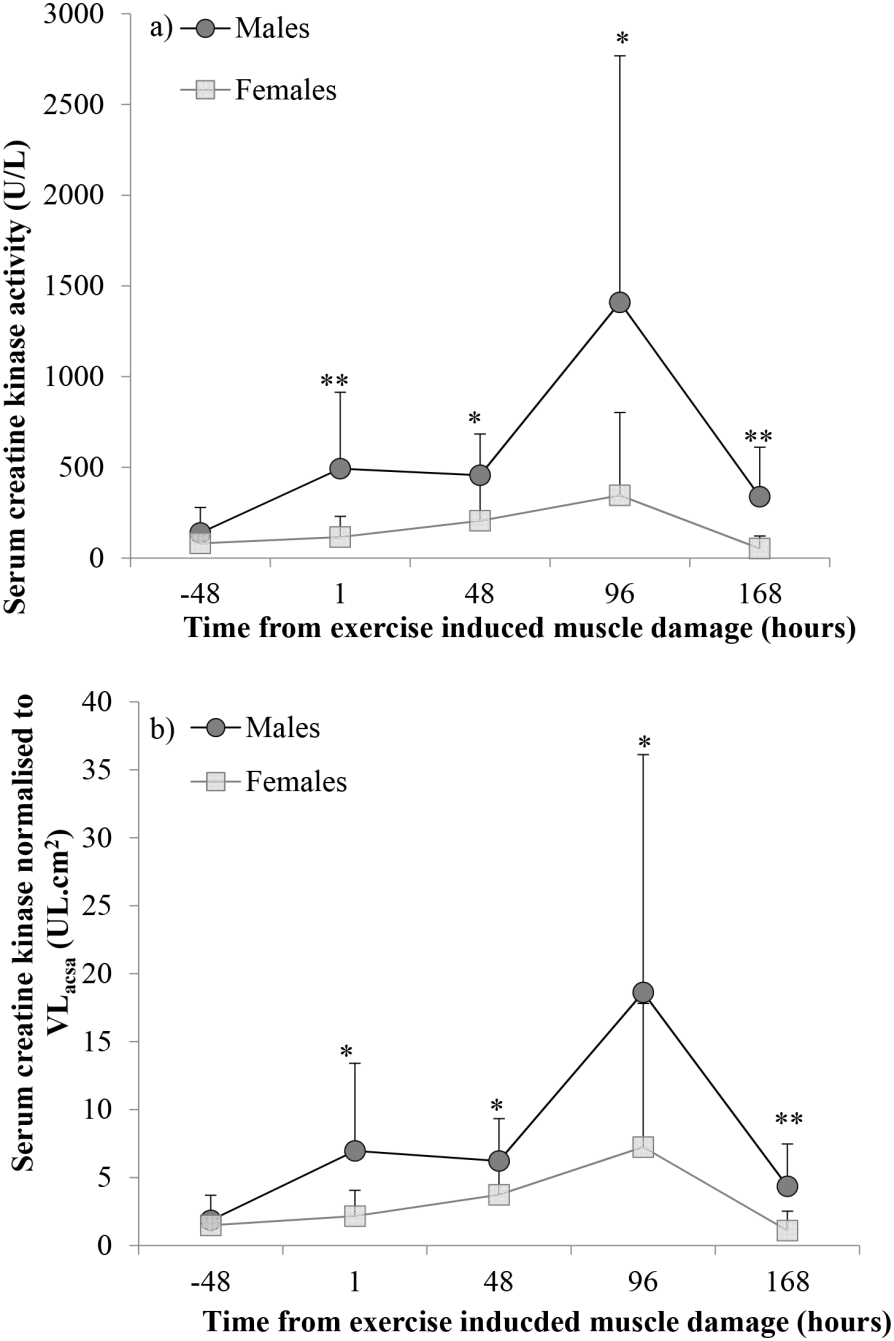
a) Serum creatine kinase levels, pre (-48 hours) exercise induced muscle damage (EIMD) and 1, 48, 96 and 168 hours post EIMD in males and females. b) Serum creatine kinase levels made relative to *Vastus Lateralis* anatomical cross-sectional area (VL_ACSA_), pre (-48 hours) exercise induced muscle damage (EIMD) and 1, 48, 96 and 168 hours post EIMD in males and females. Data is presented as means ± SD. * Males serum creatine kinase levels significantly higher than females p<0.05, ** Males serum creatine kinase levels significantly higher than females p<0.01.

Absolute CK response for males and females is illustrated in Fig 3a. A two-way mixed model ANOVA for the CK response reported a significant main effect of time (p = 0.004), group effect of sex (p = 0.0004) and interaction effect (p = 0.014). Pre-damage CK was not significantly different between males and females (138 ± 140 UL and 80.8 ± 56.7 UL, respectively, p = 0.097). Absolute peak CK was significantly higher in males compared to females (1607 ± 1247 UL and 409 ± 421 UL, respectively, p = 0.003, Cohen’s *d* = 1.05). ΔCK_ABS_ was significantly higher in males compared to females (1468 ± 1247 UL and 329 ± 441 UL, respectively, p = 0.005).

Creatine kinase response made relative to Q_ACSA_ for males and females is illustrated in Fig 3b. A two-way mixed model ANOVA for CK response made relative to Q_ACSA_ reported a significant main effect of time (p = 0.003) and a group effect of sex (p = 0.004), however there was no significant interaction effect (p = 0.112). ΔCK_ABS_ made relative to Q_ACSA_ remained significantly higher in males compared to females (18.4 ± 15.7 UL· cm^2^ and 6.84 ± 10.75 UL· cm^2^, respectively, p = 0.043).

A significant correlation was observed between maximal voluntary eccentric knee extension torque made relative to Q_ACSA_ and ΔCK_ABS_ (r = 0.635, p< 0.001), it was therefore considered as a covariate of CK. The analysis of covariance revealed a significant effect of sex on ΔCK_ABS_ when controlling for average peak maximal voluntary eccentric knee extension torque made relative to Q_ACSA_ as a covariate (p = 0.0001, Cohen’s *d* = 0.468).

### CK Correlation

Within males and females there was no significant correlation between ΔCK_ABS_ and relative patella tendon stiffness (r = -0.260, p = 0.220 and r = 0.086, p = 0.419 respectively). Using the population as a whole, there was no significant correlation between ΔCK_ABS_ and relative patella tendon stiffness (r = 0.184, p = 0.212).

Within males and females there was no significant correlation between ΔCK_ABS_ and relative change in fascicle length (males r = 0.037, p = 0.457 and females r = -0.109, p = 0.382). Using the population as a whole, there was no significant correlation between ΔCK_ABS_ and relative change in fascicle length (r = 0.251, p = 0.136).

### Muscle soreness

A two way mixed measures ANOVA for muscle soreness reported a significant main effect of time (p = 0.0004); however, there was no significant group difference of (sex, p = 0.395) or interaction effect (p = 0.759). For both males and females, peak muscle soreness occurred 48 hours post EIMD. There was no difference in peak muscle soreness in males and females (4.82 ± 2.23 and 3.75 ± 1.68, respectively, p = 0.134).

## Discussion

The aim of the current study was twofold, firstly to determine whether there is a sex difference in EIMD; and secondly, dependent on the outcome of the first aim, to establish whether tendon stiffness may explain the potential sex difference in EIMD. The three main findings of the current study are as follows: 1) maximal voluntary isometric knee extension torque loss and muscle soreness post EIMD do not differ between males and females, 2) the CK response is significantly higher in males compared to females following EIMD, and remains higher when maximal voluntary eccentric knee extension torque relative to Q_ACSA_ is considered, and 3) patella tendon stiffness and fascicle lengthening do not contribute to the sex differences in CK.

In agreement with previous literature [11,14] the current study reports no sex difference in maximal voluntary isometric knee extension torque loss post EIMD. Our findings concur with Sayers and Clarkson (14), despite the current study reporting substantially smaller maximal voluntary isometric knee extension torque loss (23% and 27% versus 54% and 60% in males and females respectively). The greater maximal voluntary contraction torque loss reported by Sayers and Clarkson (14) can be attributed to the current study exercising the lower limb rather than the upper limb, which is consistent with previous data [16]. The similar loss of maximal voluntary isometric knee extension torque in both sexes within the current study, may be supported by the findings of Stupka, Lowther (12) who, using muscle biopsies of the *Vastus Lateralis*, found no sex difference in structural damage (z-disk streaming) post EIMD. On the other hand, the current study contradicts the findings of Sewright, Hubal (7) who reported maximal voluntary contraction torque loss to be significantly higher in females compared to males immediately following 50 eccentric contractions of the elbow flexors. Interestingly, Sewright, Hubal (7) reported no further difference in torque loss post EIMD (12, 72, 96, 168 and 240 hours post EIMD). As no difference in maximal voluntary contraction torque loss was reported at any other time point, Sewright, Hubal (7) attributed the loss in maximal voluntary contraction torque immediately post EIMD to fatigue rather than EIMD. Therefore, when the confounding factor of fatigue was avoided in the current study (by measuring maximal voluntary eccentric knee extension torque loss 60 minutes after the damage protocol [26]), it can be concluded that a sex difference in torque loss post EIMD does not exist in the knee extensors. Although participant numbers (n = 22) were sufficient to report a medium effect (Cohen’s *d* = 0.41), subsequent power analysis revealed that 132 participants would be required to achieve a large effect (Cohen’s *d* ≥ 0.8). This large sample size is consistent with the large variability of maximal voluntary isometric knee extension torque loss observed in the present study, and is consistent with others. For example, within the current study maximal voluntary isometric knee extension torque loss ranged from 13–36% in males and 9–43% in females, this broad ranging response is consistent with previous EIMD research that has identified large variability associated with responders and non-responders to EIMD [16].

Despite no sex difference in maximal voluntary isometric knee extension torque loss, a significant sex difference in the CK response was reported within the current study. The CK response in both males and females followed the typical CK response post EIMD, with a peak at 96 hours post EIMD [37]. Furthermore, the elevated CK in males compared to females in the present study is consistent with the CK responses observed following both eccentric contractions in elbow flexors [7] and knee extensors [8,9]. Specifically, we have demonstrated that the acute CK response following EIMD observed by Wolf, Fragala (8) and Joyce, Sabapathy (9), persists past 24 and 48 hours (respectively), to remain significantly higher in males 168 hours post EIMD. To account for the larger muscle mass in males within the current study, and the potential confounding impact this could have, CK was also presented relative to Q_ACSA_. Creatine kinase normalized to Q_ACSA_ remained significantly higher in males. Thus suggesting that the greater muscle mass in males does not contribute to the greater CK levels post EIMD. A sex difference in CK post EIMD is not consistent with Stupka, Lowther (12) who reported no sex difference in CK following eccentric exercise of the lower limbs. Despite exercising the same muscle groups, the discrepancies between Stupka, Lowther (12) and the current study may be attributed to Stupka, Lowther (12) measuring CK 48 and 144 hours post EIMD, whereas CK is suggested to peak 72–96 hours post EIMD [37].

In further contrast to ours (CK response significantly higher in males compared to females) and those reporting no sex difference in the CK response [12], others have reported males to have lower CK and maximal voluntary isometric knee extension torque loss than females following EIMD [10]. However, it is possible that mode of EIMD (step exercise) and the omission of information on contraceptive pill use, or menstrual cycle may contribute to these differences within the literature. Therefore, the current study confirms, following maximal voluntary eccentric knee extensions, peak CK response is significantly higher in males compared to non-contraceptive pill using females.

When maximal voluntary isometric knee extension torque loss is taken as the primary marker of EIMD, the current study can conclude that there are no sex differences in EIMD however, if CK is used as the primary marker of EIMD, a sex difference is present. It remains unclear however, why a sex difference in CK has been reported within the current study. The higher CK levels following EIMD in males compared to females may be attributed to three factors, these are: 1) Significantly higher patella tendon stiffness in males, 2) greater *Vastus Lateralis* fascicle lengthening in males and 3) greater maximal voluntary eccentric knee extension torque in males. Firstly, higher circulating oestrogen levels have been reported to alter tendon properties [38], specifically lowering tendon stiffness in females [39]. In agreement with previous literature, the current study reported males to have significantly higher tendon stiffness compared to females [27]. Previously, differences in tendon properties have been suggested to explain differences in EIMD between compliant and stiff hamstrings however, the role of the tendon on EIMD has yet to be evidenced experimentally [40]. Within the current study there was no significant correlation between patella tendon stiffness and ΔCK_ABS_, in males, females or when participants were pooled by sex. Therefore, it is likely that sex differences in patella tendon properties do not explain sex differences in CK and some other factors are contributing to the reported sex difference.

Secondly, the higher CK in males may be attributed to the significantly lower fascicle lengthening in females. *In situ* animal studies the magnitude of fascicle strain, denoted by fascicle lengthening has been concluded as the main determinant of EIMD [2], *in vivo* however the role of fascicle lengthening on EIMD remains unclear [41]. Within our previous work [20] and within the current study, *Vastus Lateralis* fascicle lengthening was significantly higher in males compared to females during maximal voluntary eccentric knee extensions. Higher fascicle lengthening in the males may increase the number of fascicles extending onto the descending limb of the length tension relationship, thus increasing EIMD [42]. The current study however, showed no relationship between *Vastus Lateralis* fascicle lengthening and ΔCK_ABS_, suggesting that greater *Vastus Lateralis* fascicle lengthening in males does not explain the sex difference in CK.

Thirdly, the higher CK in males, compared to females, may be attributed to the higher maximal voluntary eccentric knee extension torque in males compared to females. *In vitro* animal studies have attributed EIMD to the magnitude of eccentric torque, within *in vivo* studies however, the association between eccentric torque and EIMD remains undetermined [43,44]. Peak maximal voluntary eccentric knee extension torque was significantly higher in males compared to females within the current study. However, when peak maximal voluntary eccentric knee extension torque was normalized to Q_ACSA_ and accounted for as a covariate, CK values remained significantly higher post EIMD in males compared to females. Therefore, although maximal voluntary eccentric knee extension torque made relative to Q_ACSA_ has an interaction with ΔCK_ABS_ (as would be expected given the greater strength and CK of the males), it did not fully account for the sex differences in CK following EIMD.

Within the current study the higher CK response in males could not be attributed to sex differences in patella tendon properties, *Vastus Lateralis* fascicle lengthening or maximal voluntary eccentric knee extension torque; it is possible therefore, that damage is equivocal between the sexes (evidenced by similar maximal voluntary isometric knee extension torque loss and muscle soreness), but in females oestrogen may suppress the release of CK. Oestrogen has a high antioxidant capacity in skeletal muscle and enhances cell membrane stability [45]. The suggested protective role of oestrogen has been investigated through the use of the oral contraceptive pill [9]. The oral contraceptive pill suppresses the natural fluctuation in oestrogen levels throughout the menstrual cycle [39]. For example, following maximal voluntary eccentric knee extension reported the CK response and the rightward shift in optimal knee angle post EIMD to be significantly greater in female oral contraceptive pill users (low oestrogen levels) compared to female non-users (high oestrogen levels). Therefore, within the current study the higher CK response in males (low oestrogen levels) compared to non-contraceptive pill using females (high oestrogen levels), may be attributed to oestrogen maintaining the structural integrity of the cell membrane within non-contraceptive pill using females. Thus, by maintaining the structural integrity of the cell membrane, oestrogen may reduce the leakage of CK into the serum and giving an impression of lower EIMD. However, the direct influence of oestrogen on sex differences in EIMD has not directly been confirmed. Although CK has historically been used as an indirect marker of EIMD [46], recently the validity of CK has been questioned [47]. In addition to large variability, it remains unclear as to whether CK is a true representation of muscle function and the magnitude of damage post EIMD, but may reflect the membrane permeability to intramuscular proteins [47]. Therefore, within the current study our findings are presented with the caveat that the elevated CK may represent a loss of membrane stability, rather than an inherent increase in EIMD *per se* [36]. In support of CK representing a loss of membrane stability, significantly higher levels of CK in males and oral contraceptive users compared to non-users has been attributed to the membrane stabilizing role of oestrogen in females [5,9].

## Conclusion

The current study can conclude that it is dependent on which marker of EIMD is used as to whether a sex difference in EIMD can be reported. It is anticipated that maximal voluntary isometric knee extension torque loss is the best indirect marker of EIMD [3], whereas it remains unclear whether CK is a true indicator of contractile disruption following EIMD [47]. Within the current study significantly higher tendon stiffness, fascicle lengthening and maximal voluntary eccentric knee extension torque in males, could not explain the lower ΔCK_ABS_ in females. Therefore, the lower CK in females may be attributed to oestrogen increasing membrane stability, thus giving the impression of greater EIMD in males [9]. Therefore, with the caveat associated with CK in mind, using maximal voluntary isometric knee extension torque loss as the primary marker of EIMD, the current study concludes a sex differences in EIMD does not exist.

## References

[1] Warren G, Hayes D, Lowe D, Armstrong R (1993) Mechanical factors in the initiation of eccentric contraction-induced injury in rat soleus muscle. The Journal of Physiology 464: 457–475. 8229813 10.1113/jphysiol.1993.sp019645 PMC1175396

[2] Lieber RL, Friden J (1993) Muscle damage is not a function of muscle force but active muscle strain. Journal of Applied Physiology 74: 520–526. 8458765 10.1152/jappl.1993.74.2.520

[3] Warren GL, Lowe DA, Armstrong RB (1999) Measurement tools used in the study of eccentric contraction-induced injury. Sports Medicine 27: 43–59. 10028132 10.2165/00007256-199927010-00004

[4] Clarkson PM, Hubal MJ (2002) Exercise-induced muscle damage in humans. American Journal of Physical Medicine & Rehabilitation 81: S52–S69. 12409811 10.1097/00002060-200211001-00007

[5] Komulainen J, Koskinen S, Kalliokoski R, Takala T, Vihko V (1999) Gender differences in skeletal muscle fibre damage after eccentrically biased downhill running in rats. Acta Physiologica Scandinavica 165: 57–64. 10072098 10.1046/j.1365-201x.1999.00481.x

[6] Borsa PA, Sauers EL (2000) The importance of gender on myokinetic deficits before and after microinjury. Medicine and Science in Sports and Exercise 32: 891 10795777 10.1097/00005768-200005000-00003

[7] Sewright KA, Hubal MJ, Kearns A, Holbrook MT, Clarkson PM (2008) Sex differences in response to maximal eccentric exercise. Medicine and Science in Sports and Exercise 40: 242 10.1249/mss.0b013e31815aedda 18202579

[8] Wolf MR, Fragala MS, Volek JS, Denegar CR, Anderson JM, et al (2012) Sex differences in creatine kinase after acute heavy resistance exercise on circulating granulocyte estradiol receptors. European Journal of Applied Physiology 112: 3335–3340. 10.1007/s00421-012-2314-z 22270483

[9] Joyce S, Sabapathy S, Bulmer AC, Minahan C (2014) The effect of prior eccentric exercise on heavy-intensity cycling: the role of gender and oral contraceptives. European Journal of Applied Physiology: 1–9. 10.1007/s00421-014-2832-y 24504652

[10] Fredsted A, Clausen T, Overgaard K (2008) Effects of step exercise on muscle damage and muscle Ca2+ content in men and women. The Journal of Strength & Conditioning Research 22: 1136–1146. 18545196 10.1519/JSC.0b013e318173db9b

[11] Rinard J, Clarkson PM, Smith LL, Grossman M (2000) Response of males and females to high-force eccentric exercise. Journal of Sports Sciences 18: 229–236. 10824639 10.1080/026404100364965

[12] Stupka N, Lowther S, Chorneyko K, Bourgeois J, Hogben C, et al (2000) Gender differences in muscle inflammation after eccentric exercise. Journal of Applied Physiology 89: 2325–2332. 11090586 10.1152/jappl.2000.89.6.2325

[13] Dannecker EA, Liu Y, Rector RS, Thomas TR, Fillingim RB, et al (2012) Sex Differences in Exercise-Induced Muscle Pain and Muscle Damage. The Journal of Pain 13: 1242–1249. 10.1016/j.jpain.2012.09.014 23182229 PMC3513404

[14] Sayers SP, Clarkson PM (2001) Force recovery after eccentric exercise in males and females. European Journal of Applied Physiology 84: 122–126. 11394240 10.1007/s004210000346

[15] Machado M, Willardson JM, Silva DP, Frigulha IC, Koch AJ, et al (2012) Creatine Kinase Activity Weakly Correlates to Volume Completed Following Upper Body Resistance Exercise. Research Quarterly for Exercise and Sport 83: 276–281. 22808713 10.1080/02701367.2012.10599858

[16] Chen TC, Lin K-Y, Chen H-L, Lin M-J, Nosaka K (2011) Comparison in eccentric exercise-induced muscle damage among four limb muscles. European Journal of Applied Physiology 111: 211–223. 10.1007/s00421-010-1648-7 20852880

[17] Roberts TJ, Azizi E (2010) The series-elastic shock absorber: tendons attenuate muscle power during eccentric actions. Journal of Applied Physiology 109: 396–404. 10.1152/japplphysiol.01272.2009 20507964 PMC2928602

[18] Roberts TJ, Konow N (2013) How tendons buffer energy dissipation by muscle. Exercise and Sport Sciences Reviews 41: 186–193. 10.1097/JES.0b013e3182a4e6d5 23873133 PMC3836820

[19] Lichtwark GA, Wilson A (2007) Is Achilles tendon compliance optimised for maximum muscle efficiency during locomotion? Journal of Biomechanics 40: 1768–1775. 17101140 10.1016/j.jbiomech.2006.07.025

[20] Hicks K, Onambele‐Pearson G, Winwood K, Morse C (2013) Gender differences in fascicular lengthening during eccentric contractions: the role of the patella tendon stiffness. Acta Physiologica 209: 235–244. 10.1111/apha.12159 23964725

[21] Brown J (1955) Urinary excretion of oestrogens during the menstrual cycle. The Lancet 265: 320–323. 10.1016/s0140-6736(55)90060-x 13234368

[22] World Medical Association (WMA). World medical association declaration of Helsinki: ethical principles for medical research involving human subjects. J Am Med Assoc. 2013;310(20):2191. 10.1001/jama.2013.281053 24141714

[23] Reeves ND, Maganaris CN, Narici MV (2004) Ultrasonographic assessment of human skeletal muscle size. European Journal of Applied Physiology 91: 116–118. 14639480 10.1007/s00421-003-0961-9

[24] Tsakoniti AE, Stoupis CA, Athanasopoulos SI (2008) Quadriceps cross-sectional area changes in young healthy men with different magnitude of Q angle. Journal of Applied Physiology 105: 800–804. 10.1152/japplphysiol.00961.2007 18556437

[25] Grosset JF, Onambele‐Pearson G (2008) Effect of foot and ankle immobilization on leg and thigh muscles' volume and morphology: A case study using magnetic resonance imaging. The Anatomical Record 291: 1673–1683. 10.1002/ar.20759 18951503

[26] Walsh L, Hesse C, Morgan D, Proske U (2004) Human forearm position sense after fatigue of elbow flexor muscles. The Journal of Physiology 558: 705–715. 15181165 10.1113/jphysiol.2004.062703 PMC1664958

[27] Onambélé GNL, Burgess K, Pearson SJ (2007) Gender‐specific in vivo measurement of the structural and mechanical properties of the human patellar tendon. Journal of Orthopaedic Research 25: 1635–1642. 17568426 10.1002/jor.20404

[28] Erskine RM, Morse CI, Day SH, Williams AG, Onambele-Pearson GL (2014) The human patellar tendon moment arm assessed *in vivo* using dual-energy X-ray absorptiometry. Journal of Biomechanics. 10.1016/j.jbiomech.2014.02.016 24612717

[29] Carolan B, Cafarelli E (1992) Adaptations in coactivation after isometric resistance training. Journal of Applied Physiology 73: 911–911. 1400055 10.1152/jappl.1992.73.3.911

[30] Lippold O (1952) The relation between integrated action potentials in a human muscle and its isometric tension. The Journal of Physiology 117: 492 12991236 10.1113/jphysiol.1952.sp004763 PMC1392416

[31] Jamurtas AZ, Theocharis V, Tofas T, Tsiokanos A, Yfanti C, et al (2005) Comparison between leg and arm eccentric exercises of the same relative intensity on indices of muscle damage. European Journal of Applied Physiology 95: 179–185. 16007451 10.1007/s00421-005-1345-0

[32] Chapman D, Newton M, Sacco P, Nosaka K (2006) Greater muscle damage induced by fast versus slow velocity eccentric exercise. International Journal of Sports Medicine 27: 591–598. 16874584 10.1055/s-2005-865920

[33] Reeves ND, Narici MV (2003) Behavior of human muscle fascicles during shortening and lengthening contractions in vivo. Journal of Applied Physiology 95: 1090–1096. 12740314 10.1152/japplphysiol.01046.2002

[34] Finni T, Ikegawa S, Lepola V, Komi P (2003) Comparison of force–velocity relationships of vastus lateralis muscle in isokinetic and in stretch‐shortening cycle exercises. Acta Physiologica Scandinavica 177: 483–491. 12648166 10.1046/j.1365-201X.2003.01069.x

[35] Bijur PE, Silver W, Gallagher EJ (2001) Reliability of the visual analog scale for measurement of acute pain. Academic Emergency Medicine 8: 1153–1157. 11733293 10.1111/j.1553-2712.2001.tb01132.x

[36] Heled Y, Bloom MS, Wu TJ, Stephens Q, Deuster PA (2007) CM-MM and ACE genotypes and physiological prediction of the creatine kinase response to exercise. Journal of Applied Physiology 103: 504–510. 17478608 10.1152/japplphysiol.00081.2007

[37] Newham D, Jones D, Edwards R (1983) Large delayed plasma creatine kinase changes after stepping exercise. Muscle & Nerve 6: 380–385. 6888416 10.1002/mus.880060507

[38] Hansen M, Miller BF, Holm L, Doessing S, Petersen SG, et al (2009) Effect of administration of oral contraceptives in vivo on collagen synthesis in tendon and muscle connective tissue in young women. Journal of Applied Physiology 106: 1435–1443. 10.1152/japplphysiol.90933.2008 18845777

[39] Bryant AL, Clark RA, Bartold S, Murphy A, Bennell KL, et al (2008) Effects of estrogen on the mechanical behavior of the human Achilles tendon in vivo. Journal of Applied Physiology 105: 1035–1043. 10.1152/japplphysiol.01281.2007 18566188

[40] McHugh MP, Connolly DA, Eston RG, Kremenic IJ, Nicholas SJ, et al (1999) The role of passive muscle stiffness in symptoms of exercise-induced muscle damage. The American Journal of Sports Medicine 27: 594–599. 10496575 10.1177/03635465990270050801

[41] Hoffman BW, Cresswell AG, Carroll TJ, Lichtwark GA (2014) Muscle fascicle strains in human gastrocnemius during backward downhill walking. Journal of Applied Physiology 116: 1455–1462. 10.1152/japplphysiol.01431.2012 23558392

[42] Morgan D (1990) New insights into the behavior of muscle during active lengthening. Biophysical Journal 57: 209–221. 2317547 10.1016/S0006-3495(90)82524-8 PMC1280663

[43] Hody S, Rogister B, Leprince P, Wang F, Croisier JL (2013) Muscle fatigue experienced during maximal eccentric exercise is predictive of the plasma creatine kinase (CK) response. Scandinavian Journal of Medicine & Science in Sports 23: 501–507. 22107069 10.1111/j.1600-0838.2011.01413.x

[44] Chapman DW, Newton MJ, Zainuddin Z, Sacco P, Nosaka K (2008) Work and peak torque during eccentric exercise do not predict changes in markers of muscle damage. British Journal of Sports Medicine 42: 585–591. 17873057 10.1136/bjsm.2007.037929

[45] Wiseman H, Quinn P (1994) The antioxidant action of synthetic oestrogens involves decreased membrane fluidity: relevance to their potential use as anticancer and cardioprotective agents compared to tamoxifen? Free Radical Research 21: 187–194. 7981789 10.3109/10715769409056569

[46] Nosaka K, Clarkson P (1996) Variability in serum creatine kinase response after eccentric exercise of the elbow flexors. International Journal of Sports Medicine 17: 120–127. 8833714 10.1055/s-2007-972819

[47] Friden J, Lieber R (2001) Serum creatine kinase level is a poor predictor of muscle function after injury. Scandinavian Journal of Medicine & Science in Sports 11: 126–127. 11252462 10.1034/j.1600-0838.2001.011002126.x

